# Serological diagnosis of chronic skin granulomas caused by wild-type or vaccine-derived rubella virus in patients with inherited HLA class I deficiency

**DOI:** 10.70962/jhi.20250103

**Published:** 2025-07-23

**Authors:** Léa Luciani, Ilad Alavi Darazam, Antoine Nougairède, Stéphane Robert, Mana Momenilandi, Jérémie Rosain, Jacinta Bustamante, Caroline Deswarte, Henri Adamski, Catherine Picard-Dahan, Nahal Mansouri, Géraldine Piorkowski, Audrey Cortes, Camille Placidi, Vincent Descamps, Felipe Suarez, Jean-Laurent Casanova, Davood Mansouri, Xavier de Lamballerie, Vivien Béziat

**Affiliations:** 1 Unité des Virus Émergents (Aix-Marseille University, Università di Corsica, IRD 190, INSERM 1207, IRBA), Marseille, France; 2 Department of Infectious Diseases, Loghman Hakim Hospital, Shahid Beheshti University of Medical Sciences, Tehran, Iran; 3 C2VN, AMUTICYT Core Facility, INSERM, INRA, Aix-Marseille University, Marseille, France; 4 Laboratory of Human Genetics of Infectious Diseases, Necker Branch, INSERM, Necker Hospital for Sick Children, Paris, France; 5 St. Giles Laboratory of Human Genetics of Infectious Diseases, Rockefeller Branch, The Rockefeller University, New York, USA; 6 Imagine Institute, University of Paris-Cité, Paris, France; 7 Department of Dermatology, Pontchaillou Hospital, Rennes, France; 8 Department of Dermatology, Bichat Hospital AP-HP, Paris, France; 9 Division of Pulmonary Medicine, Department of Medicine, Lausanne University Hospital, University of Lausanne, Lausanne, Switzerland; 10 Howard Hughes Medical Institute, the Rockefeller University, New York, USA; 11 Pediatric Hematology-Immunology and Rheumatology Unit, Necker Hospital for Sick Children, AP-HP, Paris, France; 12 Clinical Tuberculosis and Epidemiology Research Center, National Research Institute of Tuberculosis and Lung Diseases, Shahid Beheshti University of Medical Sciences, Tehran, Iran

## Abstract

Patients with TAP1/TAP2 deficiency develop rubella-associated skin granulomas due to impaired HLA-I antigen presentation. Markedly elevated rubella IgG levels may support diagnosis when PCR is unavailable. These findings also underscore the specific vulnerability of such patients to live-attenuated rubella vaccination.

Bare lymphocyte syndrome type 1, also known as human leukocyte antigen (HLA) class I deficiency, is associated with very low HLA-I expression (1–3% of the normal values). This autosomal recessive disease is caused by biallelic variants in either *TAP1*, *TAP2*, *TAPBP*, or *B2M* genes (1). TAP1 and TAP2 form the transporter associated with the antigen processing (TAP) complex essential for peptide translocation from the cytosol to the endoplasmic reticulum, and their presentation on HLA-I. Tapasin, encoded by *TAPBP*, connects empty HLA-I with the TAP complex. The β2-microglobulin, encoded by *B2M*, is a chaperone molecule stabilizing HLA-I expression. Onset of clinical disease in patients with TAP1 or TAP2 deficiency usually occurs in late childhood or adulthood. Their clinical phenotype ranges from asymptomatic to bacterial infections, including sinusitis, chronic respiratory tract inflammation, and granulomatous skin lesions (50% of individuals). A similar phenotype was described in two patients with β2-microglobulin deficiency. Two patients with tapasin deficiency were shown to have lung bacterial infections and herpes zoster, but no granulomatous skin lesions. The origin of the granulomatous skin lesions remained mysterious for a long time, as no pathogens were identified in the lesions. Recent reports in two patients suggest that the granuloma of patients with inherited HLA-I deficiencies may be driven by rubella virus (RuV) (2, 3). We therefore tested the hypothesis that the granulomas observed in four patients with TAP deficiency were associated with RuV.

P1 and P2 were related Iranian women born from consanguineous parents. Their clinical data were described elsewhere (4), as I.4 and II.2, respectively. Briefly, P1 was a 62-year-old woman with hypopigmented ulcerative patches and plaques with itching, erythema, and severe edema involving the lower limbs since her late twenties. She died in 2023 due to multiple complications associated with her underlying immunodeficiency, including skin infections. P2 was the 48-year-old cousin of P1. Her medical history was unremarkable except for chronic skin lesions on her right leg and foot for more than 20 years. The lesions were consistent with necrotizing granulomatous inflammation. By whole-exome sequencing (WES) of P1 and P2, a homozygous loss-of-function variant was identified in *TAP2* (NM_001290043.2; c.979_983delinsGGGG; p.Arg327Glyfs*53). P3 was a 54-year-old man of Kurdish ancestry born to consanguineous parents (5). He was living in France since his early 30s. He suffered from chronic lung infection, leading to bronchiectasis, and aspergillus infection. He was diagnosed with common variable immunodeficiency and received subcutaneous immunoglobulin. He developed granulomatous skin lesions on both arms and the left leg since his early twenties. His skin lesions were complicated with four squamous cell carcinomas (SCC), all completely surgically removed. P3 developed a fifth metastatic SCC and died in 2023. By WES, he was shown to carry a homozygous premature stop codon in *TAP1* (NM_000593.6, c.1132C>T; p.Arg378*). P4 is a 22-year-old French woman born to non-consanguineous parents (6). Briefly, she had granulomatous inflammatory skin lesions since the age of 4 and recurrent lung bacterial infection since the age of 11, leading to bronchiectasis. By WES, she was found to carry a homozygous premature stop codon in *TAP1* (c.1699A>T; p.Lys567*). Similar to P2 (4) and in line with HLA-I deficiency, the immunophenotyping of P3 and P4 showed a marked decrease in naïve CD8 T cells and a CD4/CD8 ratio in naïve T cells >100 times higher than normal values (Fig. 1, A–F). Of note, the counts of MAIT and iNKT cells of P3 and P4 are within the normal range (Fig. 1 G), contrasting with our previous report of two TAP2-deficient patients, including P2 (4).

**Figure 1.**
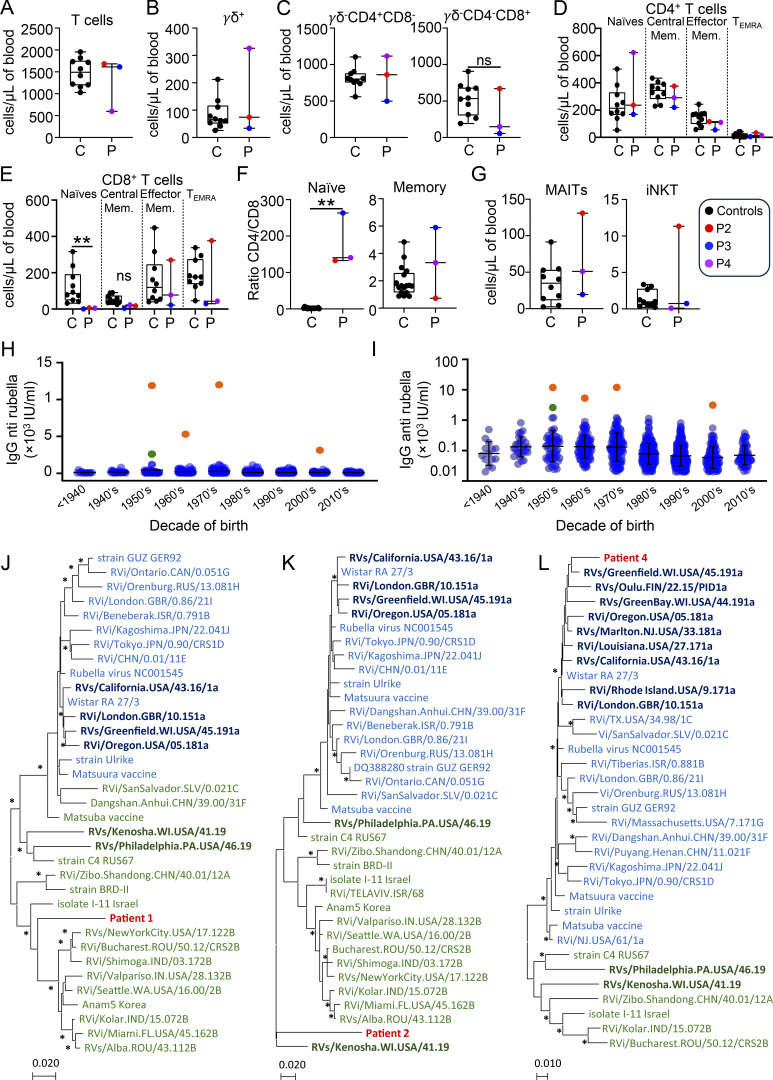
**T cell immunophenotyping and rubella infection in HLA-I deficiency. (A)** CD3^+^ T cell counts. **(B)** CD3^+^TCR*γ*δ^+^ T cell counts. **(C)** CD3^+^TCR*γ*δ^−^CD4^+^CD8^−^ and CD3^+^TCR*γ*δ^−^CD4^−^CD8^+^ T cell counts. **(D)** Counts of naïve (CD45RA^+^CCR7^+^), central memory (CD45RA^−^CCR7^+^), effector memory (CD45RA^−^CCR7^−^), and TEMRA (CD45RA^+^CCR7^−^) CD4 T cells. **(E)** Counts of naïve (CD45RA^+^CCR7^+^), central memory (CD45RA^−^CCR7^+^), effector memory (CD45RA^−^CCR7^−^), and TEMRA (CD45RA^+^CCR7^−^) CD8 T cells. **(F)** Ratio CD4/CD8 in naïve (left) and memory (right) CD3^+^TCR*γ*δ^−^ T cells. **(G)** MAIT (CD3^+^CD161^+^TCRva7.2^+^) and invariant NKT (CD3^+^iNKT^+^) unconventional ⍺β T cell counts. **(H and I)** Representation of anti-rubella IgG titers in IU/ml and (I) logarithmic scale with representation of the mean and standard deviation according to the decade of birth of the subjects. The orange dots represent the four patients of the present study (P1–P4 by chronological order of birth). The green dot corresponds to a recently reported patient with rubella granuloma and ITK deficiency (7). The blue dots correspond to the serological titers of all patients who had a rubella serology prescription at the Assistance Publique Hôpitaux de Marseille (excluding the obstetrics department) from 01/01/2022 to 31/12/2022 with negative IgM and detectable IgG (>20 IU/ml). **(J)** Phylogenetic analysis of rubella partial genome sequence of strain from patient 1 (PV855062). A total of 2,696 nucleotide sequences (positions 230–472, 636–861, 1087–1316, 3874–4125, 4522–4987, 5106–5344, 5528–5744, 6916–7160, 8328–8572, and 8741–9073 on reference NC_001545). **(K)** Phylogenetic analysis of rubella partial genome sequence of strain from patient 2 (PV855063). A total of 519 nucleotide sequences (positions 8569–9088 on reference NC_001545). **(L)** Phylogenetic analysis of rubella partial genome sequence of strain from patient 4 (PV855064). A total of 3,566 nucleotide sequences (positions 239–473, 1506–1680, 1794–2044, 3271–3668, 3936–4172, 4376–4606, 4802–5277, 5660–5900, 6915–7142, 7835–7887, and 8530–9570 on reference NC_001545). For all patients, nucleotide sequences were obtained directly from skin biopsies. Phylogenetic trees were constructed using MEGA7 software with the maximum likelihood method and the Tamura–Nei model. Bootstrap values (calculated with 1,000 replicates) lower than 75% are not shown, and those above 75% are indicated by an asterisk. RuV sequences belonging to clade 1 are shown in blue. The sequences in bold and darker blue correspond to RuVs responsible for chronic infections derived from the Wistar RA27/3 vaccine strain. Sequences in green belong to clade 2. The two sequences in bold and dark green correspond to two cases of chronic infection from wild-type strains. The patient’s sequences are in dark red.

No pathogens were detected in patients’ granulomatous lesions using standard microbiological cultures. The skin lesions did not improve under antibiotics, antifungal and anti-mycobacterial drugs (all patients), ustekinumab (P3), infliximab (P3 and P4), hydroxychloroquine (P3 and P4) or betamethasone (P3), or interferon γ-1b (P4). Based on the recent identification of RuV in the lesions of one TAP1 and one TAP2 patient (2, 3), we hypothesized that their lesions were due to rubella infection. All patients were IgM negative and strongly IgG positive for rubella, indicating past infection. We compared their IgG levels with a reference panel from patients tested at the Assistance Publique des Hôpitaux de Marseille in 2022. Only those with the same serological profile (IgG positive, IgM negative) were included. Patients from gynecology/obstetrics departments were excluded due to frequent revaccination in women planning pregnancy. In total, we obtained 1,023 patients aged from 3 to 87 years, with an M/F sex ratio of 0.4. Because of different exposure to RuV by date of birth (natural circulation, one-dose or two-dose vaccination), the results presented in Fig. 1, H and I, are stratified by decade of birth. The average anti-rubella IgG level varies between 78 and 222 IU/ml with standard deviations between 78 and 299 IU/ml, depending on the decade of birth. However, the four TAP1-mutated patients have considerably higher rubella IgG titers: 11,900 IU/ml, 12,000 IU/ml, 5,300 IU/ml, and 3,130 IU/ml, respectively, for patient 1, 2, 3, and 4. Thus, the four patients had strong antibody responses to RuV infection, with IgG levels 30–60 times higher than their respective age-matched control group, which may suggest a continuous antigenic stimulation of the antibody response.

Next, the presence of RuV was tested by PCR on skin biopsies. We first performed PCR assays on skin biopsies in the absence of reverse transcription. The lack of amplification ruled out the presence of RuV DNA forms, indicating the absence of viral integration into the host genome (data not shown). Instead, three out of four patients had a positive PCR after reverse transcription. The cycle threshold was 35, 37, and 31 for P1, P2, and P4, respectively. P3, despite several attempts at RNA concentration and different systems tested, remained negative, possibly reflecting inappropriate sampling. Using a combination of conventional sequencing (Sanger) and next-generation sequencing (S5 Ion torrent) approaches, we obtained for P1 a total sequence of 2,696 nucleotides (i.e., almost one third of the genome), which reveals that the virus is a wild-type strain and would belong to clade 2 (Fig. 1 J). For P2, we only managed to obtain a small sequence of 519 nucleotides, but this is located in the region for the E1 envelope protein, which is the one recommended by the WHO to perform rubella genotyping. The results are interesting because the virus appears to be distant from other sequence strains available in GenBank, and the closest virus strain is MT249313 RVs/Philadelphia.PA.USA/46.19, which is one of the three strains deposited to date in GenBank from a chronic non-vaccine rubella infection that is also itself divergent from anything currently known (Fig. 1 K). These data corroborate the fact that P1 and P2 were not vaccinated and given their decade of birth were probably exposed to a natural RuV infection. The probable presence of clade 2 is also consistent with the geographical origin of these patients from the Middle East. For P4, we obtained a sequence of 3,566 nucleotides (36% of the genome). This patient was younger and had been vaccinated with the Wistar RA27/3 strain. We confirm that the vaccine strain is found in the lesions and a genetic drift is observed as in children with primary immune deficiency for whom the complete genome sequence of vaccine-derived strain has been deposited in GenBank (Fig. 1 L). The three partial nucleotide sequences of P1, P2 and P4 are available on GenBank under accession numbers PV855062, PV855063 and PV855064 respectively.

In this study, we report chronic cutaneous granulomatous lesions associated with RuV infection in four patients with TAP deficiency. All patients were seropositive for rubella, with antibody levels strongly higher than other individuals tested in our center over a year. We identified the wild-type RuV (P1 and P2) or a vaccine-derived strain (P4). A definitive diagnosis of cutaneous rubella granuloma in P3 cannot be established due to the negative PCR result. However, there is a strong diagnostic presumption based on a similar clinical presentation, TAP deficiency, and an exceptionally high IgG titer. Unfortunately, to date, no therapeutic approach was consistently successful against chronic rubella infection, except hematopoietic stem cell transplantation, including in one patient with concomitant TAP1 and TAP2 deficiency (8). Given the dreadful evolution of the disease over the years, with a major impact on the patients’ life quality and expectancy, such therapeutic option may be considered. Nevertheless, the long-term outcome of such approach is uncertain, as HLA-I deficiency would not be corrected in thymic stromal cells and other non-hematopoietic cells.

In conclusion, in the absence of viral PCR on a skin sample, rubella serology showing extremely elevated titers is suggestive of the diagnosis in patients with unexplained skin granulomas. Serology appears to be a rapid and effective diagnostic tool, provided that the tested patient has an intact humoral immunity. Future studies should explore the serological profiles of individuals with inherited HLA class I deficiency who have been vaccinated or infected, yet did not develop cutaneous granulomas. With two previous isolated reports (2, 3), our findings suggest that inherited TAP1 and TAP2 deficiencies specifically predispose to skin RuV infection; they should not be vaccinated with live-attenuated rubella vaccines.

## Data Availability

The data are available from the corresponding author upon reasonable request.
